# Flexor Digitorum Superficialis and Flexor Digitorum Profundus with separated sheaths: a new normal variation in human

**Published:** 2015-12-30

**Authors:** Seyed Mokhtar Esmaeilnejad-Ganji, Behnam Baghianimoghadam

**Affiliations:** Department of Orthopedic Surgery, Shahid Beheshti Hospital, Babol University of Medical Sciences, Babol, Iran

**Keywords:** Flexor digitorum superficialis, flexor digitorum profundus, hand, tendon

## Abstract

**Case description:**

A 25 years old man presented with a laceration on radial side of proximal phalanx of 4^th^ finger (zone II flexor) which was due to cut with glass.

**Clinical findings:**

The sheaths of Tendons of flexor digitorum sperficialis and profundus were not the same and each tendon had a separate sheath.

**Treatment and outcome:**

The tendons were reconstructed by modified Kessler sutures, after 15 months the patient had a 30 degrees of extension lag even after physiotherapy courses.

**Clinical relevance:**

This is the first reported of such normal variation in human hand tendon anatomy.

## Abstract (es)

**Descripción del caso:**

Se presentó un hombre de 25 años con una laceración en la parte radial de la falange proximal del cuarto dedo de la mano (zona flexor II) causada por el corte con un vidrio.

**Hallazgos clínicos:**

Las cubiertas de los tendones del flexor digitorum sperficialis y profundus estaban separadas en diferentes cubiertas.

**Tratamiento y resultado:**

Los tendones se reconstruyeron por la suturas modificadas de Kessler. Después de 15 meses el paciente presentó una pérdida del 30% en la extensión , aun después de la fisioterapia.

**Relevancia clínica:**

Es el primer reporte de la variación en la anatomía de la mano.

## Introduction

All digital flexors have origin from the medial epicondyle, anterior of radius, ulna and interosseous membrane. These tendons become tendentious in distal third of forearm and pass through the osteofibrous tunnel named carpal tunnel. In carpal tunnel the most superficial component is median nerve which is tend to locate on radial side of canal 1. Tendons of flexor digitorum sperficialis (FDS) and profundus (FDP) (with flexor policis longus) are located deeper respectively 1,2. Tendons of FDS and FDP are enclosed in a common synovial sheath, the ulnar bursa which is extended from wrist proximally to hand distally; and continues to synovial bursa of fifth finger. Flexor policis longus has its own specific sheath (radial bursa) 3. 

When after entering the palm of hand, the flexor tendons fans out to their respective digits. Tendons of flexor digitorum sperficialis are superficial to the FDPs 4. When tendons enter the fingers, they invested in a strong osteofibrous canal. Ligamentous part is named the tendon sheath which is lined by synovial sheath (digital bursa) which is reflected on the contained tendon. Different region of fibrous sheath are thickened to form pulleys (annular or cruciate). Within the sheaths, FDS and FDP are tethered to phalanxes by expressions of mesotendons named vincula brevia and longa which also carry the blood supply to tendons 5-7.

There is no previous report on separated sheath of FDS and FDP in human. We in this case report present a patient with separated FDS and FDP sheath, a new variation of hand finger flexor tendons (FDS and FDP) which is not reported in human anatomy.

## Case description

A 25 years old man presented with a laceration on radial side of proximal phalanx of 4^th^ finger (Zone II) which was due to cut with glass. During initial examination the FDS and FDP seemed to be cut. Then patient was admitted for flexor tendon repair. 

During surgery the primary wound was extended by Z plasty which flaps was retracted and then we tried to reconstruct the both FDS and FDP as patients was an intern in general medicine and interested in surgery and also was a piano player which made him a high demand case.

To find the retracted tendon we tried to find the cut tendons in distal and proximal canals. During try to find tendons, we had to extend the field of surgery. Surprisingly we saw that the proximal parts of FDS and FDP are separated. At first we extended the field more as were suspicious to retraction of the FDP from its normal canal and extruded out of canal at proximal site. But we found that the sheaths of FDP and FDS are not the same and each tendon have separate sheath. The tendons were repaired by Kessler and running sutures. The Klinerth system 7 was applied for the patient and passive flexion with active extension was started from the fifth day after surgery. Patient was followed weekly and the process of rehabilitation was screened. After 23 days the splint was removed and physiotherapy was started. During 15 month after surgery about 30 degrees of extension lag was remained which was not restored.

**Figure 1.**
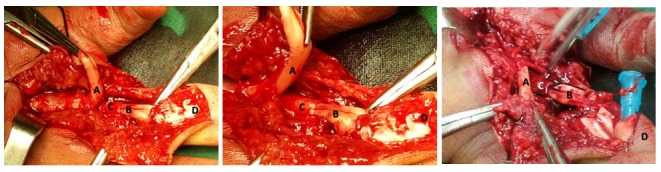
The Flexor Digitorum Profundus (A) and Flexor Digitorum Superficialis (B), with separate sheaths (C) in the palm of hand at zone II region. D shows the base of 4^th^ finger. Normally Each FDS (B) and FDP(A) tendons of fingers runs together among a tendon sheath into the fibro-osseous canal of each digit (D).In this variation the FDP and FDS have separated sheath (C).

## Discussion

Based on normal anatomy which is mentioned in all textbooks and articles focused on human anatomy and hand surgery, the FDS and FDP originate in the forearm, at the medial epicondyle of the elbow. They, along with flexor pollicis longus (FPL) and the median nerve, and go through the carpal tunnel at the wrist and enter the palmar surface of the hand. Then FDS and FDP send individual tendons to the index, long, ring, and small fingers. Each FDS and FDP tendons of fingers runs together among a tendon sheath into the fibro-osseous canal of each digit. This specific canal is formed by the metacarpals and phalanges, and by the pulley system and tendon sheath 1-7. 

Within literatures, some anomalies of FPL are described. The most frequent anomaly is an additional connection between FPL and FDP of index. Also there are reports on tendon sheath of FPL which is enclosed to FDP II with thick adherence 7-9.

Our search on other species especially apes (Pan, Gorilla, and Pongo) revealed the mostly is on the place for fanning of FDP (In forearm or hand). But such a variation which is described in our case is not specifically described in other species too 10,11.

Our patients was pianist, because of ethical considerations we was not able to wider incisions and explore of other digit as it may be present in other finger flexors too. As this is the first reported case of this variation, the clinical significance of such an anatomy is not clear, but possibly the separated FDP possibly could help in delicate activities as FDP theoretically has more free space for excursion. 

Based on our knowledge there is no other reported variation in sheath of FDP and FDS in hand. Of course there are several variations on the FDS previously but such a variation which is described in our case is not reported before. 
